# Supplementary material for: A self-eliminating allelic-drive reverses insecticide resistance in Drosophila leaving no transgene in the population
Source: Nat Commun. 2024 Nov 17;15:9961. doi: 10.1038/s41467-024-54210-4 (PMC11570635; doi:10.1038/s41467-024-54210-4)
Supplement: Supplementary file 1 — Supplementary Information [file 41467_2024_54210_MOESM1_ESM.pdf]

Supplementary Information

A self-eliminating allelic-drive reverses insecticide resistance in *Drosophila* leaving no transgene in the population

Ankush Auradkar[1,2], Rodrigo M. Corder[3,4], John M. Marshall[4,5] and Ethan Bier[1,2*]

[1]Department of Cell and Developmental Biology, University of California, San Diego, 9500 Gilman Drive, La Jolla, CA 92093-0335, USA

[2]Tata Institute for Genetics and Society-UCSD, 9500 Gilman Drive, La Jolla, CA 92093-0335, USA

[3]Department of Parasitology, Institute of Biomedical Science, University of São Paulo, São Paulo, SP, 05508-000, Brazil

[4]Divisions of Biostatistics and Epidemiology - School of Public Health, University of California, Berkeley, CA, 94720, USA

[5]Innovative Genomics Institute, Berkeley, CA, 94720, USA

*Corresponding author: ebier@ucsd.edu

The PDF file includes:

Supplementary notes
Supplementary Figure 1 to 7
Supplementary Table 1 to 4

Supplementary notes

Model description

The dynamics of the self-eliminating allelic drive were modeled considering discrete generations to account for the non-overlapping nature of the experiments. We consider a simplified framework in which we represent all the main genotype features studied and expected to occur in the experiment by 5 different letters, as indicated in Supplementary Table 1. In this framework, we modeled the X chromosome(s) in each fly by indicating the status of the *para* locus (F for *para*^{1014F}, L for *para*^{1014L} and N for male lethal NHEJ at *para*^{1014F}), and the *yellow* locus (C for the composed system vasaCas9+gRNA-F+DsRed and Y for the wild type *yellow* locus). This simplified framework (Supplementary Table 1) allows us to track genotypic and phenotypic frequencies throughout non-overlapping generations, according to sex, as shown in Supplementary Table 2.

The cages were seeded at phenotypic frequencies of (i) 1:3, with 25% of the flies carrying *e-Dr, para*^{1014L} (15 males represented by CL and 15 females represented by $CCLL$), and 75% of the flies carrying y^{+},w^{-}, *para*^{1014F} or y^{-},w^{-}, *para*^{1014F} (45 males YF and 45 females $YYFF$), and (ii) 1:1, with 50% of flies *e-Dr, para*^{1014L} (30 males CL and 30 females $CCLL$) and 50% of flies y^{+},w^{-}, *para*^{1014F} or y^{-},w^{-}, *para*^{1014F} (30 males YF and 30 females $YYFF$).

We modeled allelic-drive action by assuming that *para*^{1014F}/*para*^{1014L} females carrying the e-Drive system might convert *para*^{1014F} into *para*^{1014L} in the germline according to the rate r_C, or into lethal *para*^{1014F}, here denominated as *para*^{lethal}, according to the rate r_{lethal}. We assume that *para*^{lethal} male and *para*^{lethal}/*para*^{lethal} female flies are inviable and do not develop to the adult stage. We also assume that *para*^{1014F} males, *para*^{1014F}/*para*^{1014F} and *para*^{lethal}/*para*^{1014F} females are inviable if they carry the e-Drive system since there is no "template allele" to accurately repair the *para*^{1014F} locus in response to cleavage by the e-Drive system. In these cases, we hypothesized that *para*^{1014F} alleles will be targeted by the e-Drive system consecutively and eventually will become *para*^{lethal}. We also define the parameter r_L to account for those events in which the drive did not act at the *para*^{1014L} target site in the germline. In this framework, we assume that $r_C + r_{lethal} + r_L = 1$.

At each experimental generation, we assumed that flies are well mixed and females mate only once. Because the *yellow* locus and the *para* locus are far from each other on the X chromosome, we also assumed that their inheritance are independent. The genotypic frequency of each progeny, assumed to be composed of 50% male and 50% female, is obtained based on the genotypic frequency of the previous adult generation. We assumed that male flies carrying the e-Drive system are less competitive to mate according to factors $m_{\text{e-Dr}}$ ($0 < m_{\text{e-Dr}} \leq 1$, with $m_{\text{e-Dr}} = 1$ indicating equally competitive as those flies not carrying the e-Drive) and $m_{\text{e-Dr,hover}}$ ($0 < m_{\text{e-Dr,hover}} \leq 1$), for the allelic self-eliminating and hover mode experiments, respectively. Since the first generations observed in the multi-generational cages experiment show a greater fraction of $para^{1014L}$ alleles than would be expected by simple chromosome segregation, we confirmed the hypothesis that $para^{1014F}$ males carry a fitness cost, [16] and Sup. Figure 4, according to the factor f_{F} ($0 < f_{\text{F}} \leq 1$, with $f_{\text{F}} = 1$ indicating no fitness cost), which weights down the relative progeny numbering of all viable $para^{1014F}$ males.

As for $para^{1014F}$ females, we tested the hypothesis that $para^{1014F}$ homozygous are either less viable and/or less fertile according to factors $f_{\text{DD,viab}}$ ($0 < f_{\text{DD,viab}} \leq 1$, with $f_{\text{DD,viab}} = 1$ indicating no reduced viability) and $f_{\text{DD,fert}}$ ($0 < f_{\text{DD,fert}} \leq 1$, with $f_{\text{DD,fert}} = 1$ indicating no reduced fertility), respectively. In accordance with previous reports [16, 23], we also tested the effects of maternally transmitted Cas9/gRNA ribonucleoprotein complexes that more efficiently cleave and/or convert the targeted $para^{1014F}$ allele. To this end, we considered that mothers carrying the e-Drive system may influence the $para^{1014F}$ allele(s) in early stages of the progeny, according to the rate md, changing the phenotype of these individuals. Following the same rationale of the action of the e-Drive in germline, if the mother carries the e-Drive system, we assumed that, for all the progeny and according to the rate md, (i) $para^{1014F}$ males and $para^{1014F}/para^{1014F}$ females may become inviable, (ii) the $para^{1014F}$ allele of $para^{1014F}/para^{1014L}$ females may be converted to $para^{1014L}$, and (iii) $para^{1014F}/para^{\text{lethal}}$ females may become inviable. We summarize description and estimation of parameters in Supplementary Table 3.

We used the same mechanism described above to model the hover mode experiment. However, because no or low fitness costs associated to the e-Drive system are expected for this experiment,

we tested the hypothesis of neutral fitness for the parameter associated with the e-Drive system, that is, $m_{\mathrm{e-Dr,hover}}$ equal to 1.

We used parameter values inferred by the model fitting to simulate control experiments, in which the e-Drive was implemented without vasa-Cas9. We assumed the same values for the reduced fertility of $para^{1014F}$ homozygous females $f_{FF,fert}$, and the mating competition factor for males carrying e-Drive $m_{\mathrm{e-Dr}}$. We also assumed neutral mating competition $m_{\mathrm{e-Dr,hover}} = 1$. For these experiments, however, since the e-Drive was implemented without vasa-Cas9, we assumed that rates r_C, r_{lethal} and md are 0 (Supplementary Table 3) and all individuals are viable. Simulations are reported in Sup. Figure 6.

Model fitting

The model fitting was carried out considering the likelihood of the sex-specific DsRed and $para^{1014L}$ frequencies observed in both self-elimination and hover mode cage experiments given the model output. To this end, we used Markov chain Monte Carlo (MCMC) methods to obtain posterior estimates and 95% credible intervals for each parameter (Supplementary Table 3), considering that the measured DsRed and $para^{1014L}$ frequencies can be represented by binomial distributions with probabilities given by the simulated frequencies at each generation. No prior distributions were used in the model fitting. All simulations were performed and analyzed in R. Results for the 1:3 and 1:1 ratio experiments are presented in Sup. Figure 4 and 5, respectively.

Stochastic simulations

Simulated model trajectories were generated using a stochastic implementation of the discrete-generation model. At each generation, the offspring genotypic frequency is assumed to follow a multinomial distribution informed by the composite mated female genotype and the inheritance patterns of the allelic-drive system, modified by the genotype-specific fertility and fitness costs. Female and male adults from each generation are then sampled to seed the next generation, with sample size defined by the number of flies sampled in each generation (50). Results for the 1:3 and 1:1 ratio experiments are presented in Sup. Figure 4 and 5, respectively.

Model selection

To assess the statistical favorability of each biologically assumption, we initially optimized the model accounting for all the mechanisms (that is, all parameters) described in the Model description and assumed that the e-Drive system was fitness-neutral in the "hover" mode experiment. Herein, we refer to this configuration as the “standard model”. Next, we tested the hypothesis that males carrying the e-Drive system are not fitness-neutral and are less competitive to mate also in the hover mode (parameter $m_{\mathrm{e-Dr,hover}}$). The Akaike information criterion (AIC) values obtained after model optimization suggest it is more likely that males carrying the e-Drive system are also less competitive to mate in the hover mode (Supplementary Table 4) with respect to those not carrying the e-Drive system.

We next tested if the mechanisms introduced by each model parameter (Supplementary Table 3) would favor the model fit to the experimental data. To this end, we computed the likelihood of observing the data and the AIC values given the standard model, e-Drive male competitiveness in the hover mode, and excluding one by one of the parameters described in the Model description. The minimum AIC value was obtained for the model configuration which does not account to the reduced viability of homozygous *para*^{1014F} females ($f_{FF,viab}$; Supplementary Table 4).

We next excluded the parameter $f_{FF,viab}$ and, similarly, tested the favorability of simpler models by excluding one by one of the remaining parameters and computing the likelihood of observing the data and AIC values. The AIC values favors the model configuration in which *para*^{1014F} males do not carry a fitness cost, i.e., by excluding parameter f_{F}. No other simpler configuration was found according to the AIC values. Therefore, the best-fitting model (Supplementary Table 4) is defined by 6 parameters with values and 95%CrI as presented in Supplementary Table 3.

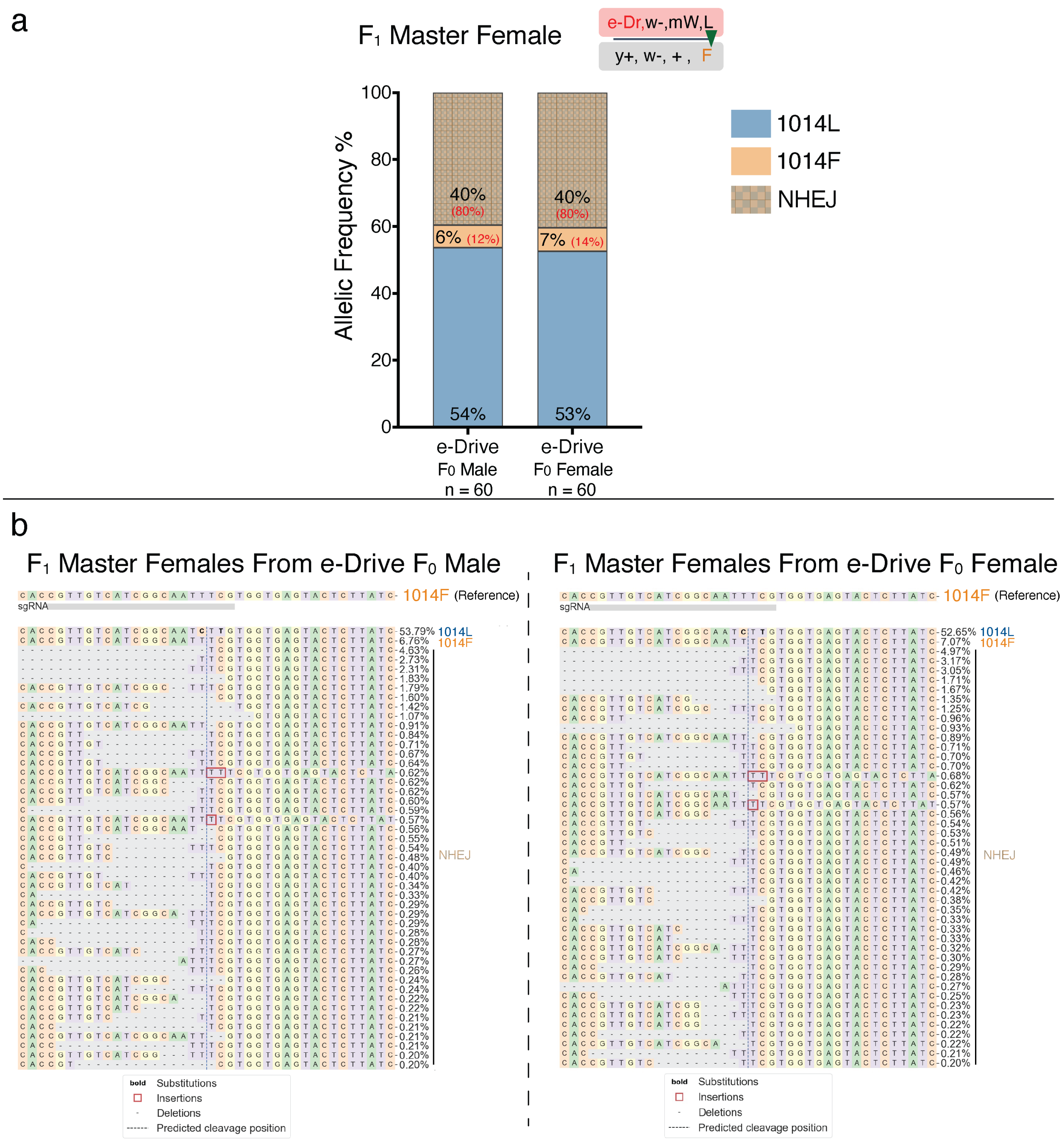

Supplementary Figure 1.

Analysis of germline transmitted *para* alleles in F_2 females. (a) *para* allelic frequency (1014L, 1014F, NHEJ) calculated from amplicon sequencing data for F_1 master females from cross scheme shown in Figure 2c-d. We estimated the NHEJ percentages on the receiver chromosome assuming that the receiver chromosome accounts for ~50% of the reads according to the formula 2X NHEJ%,

indicated in red. **(b)** Representative data from amplicon sequencing of F_1 master females from e-Drive carried by F_0 males (left panel) or females (right panel).

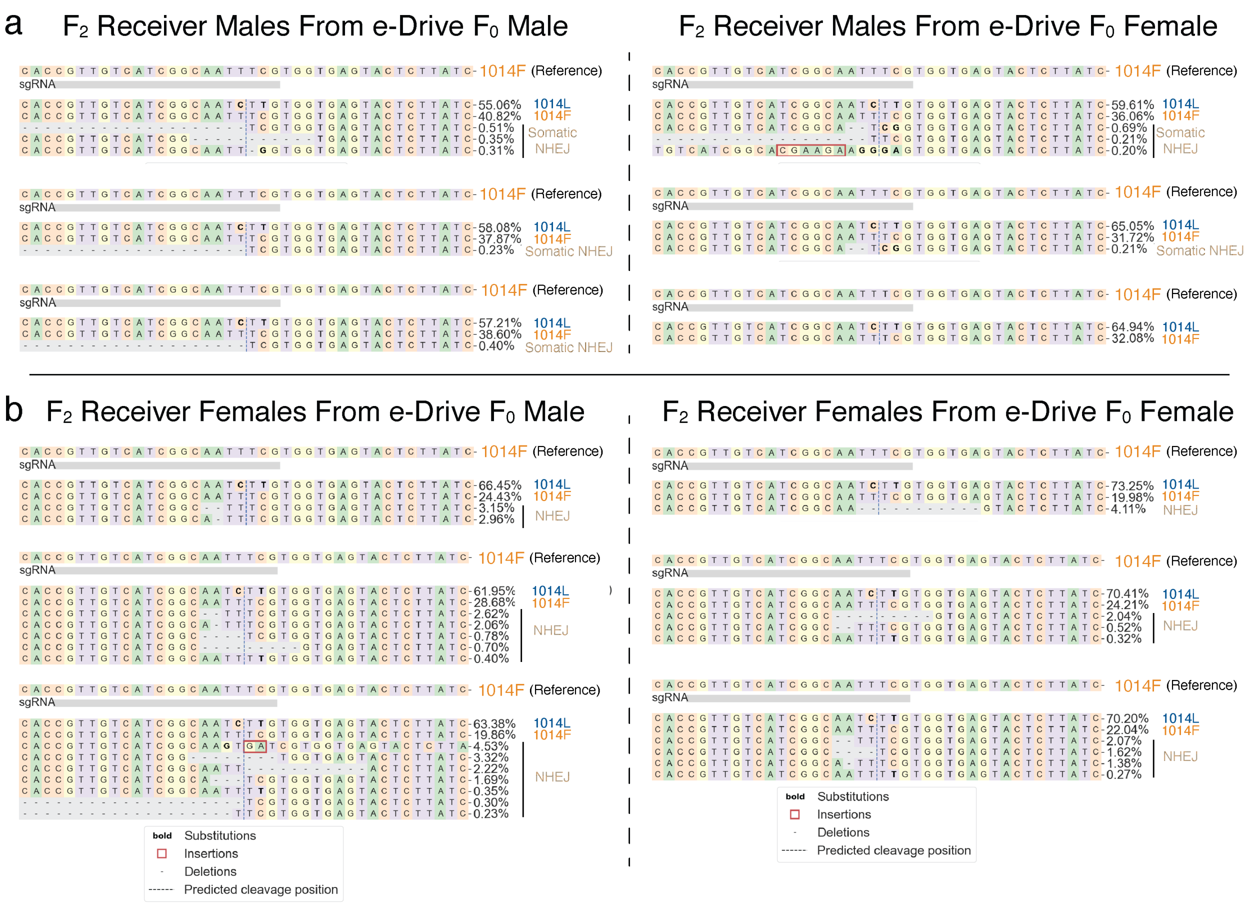

Supplementary Figure 2.

Analysis of germline transmitted *para* alleles in F$_2$ males. Representative data from amplicon sequencing of F$_2$ receiver males **(a)** and females **(b)** from e-Drive carried by F$_0$ males (left panel) or females (right panel) from cross scheme shown in Figure 2c-d.

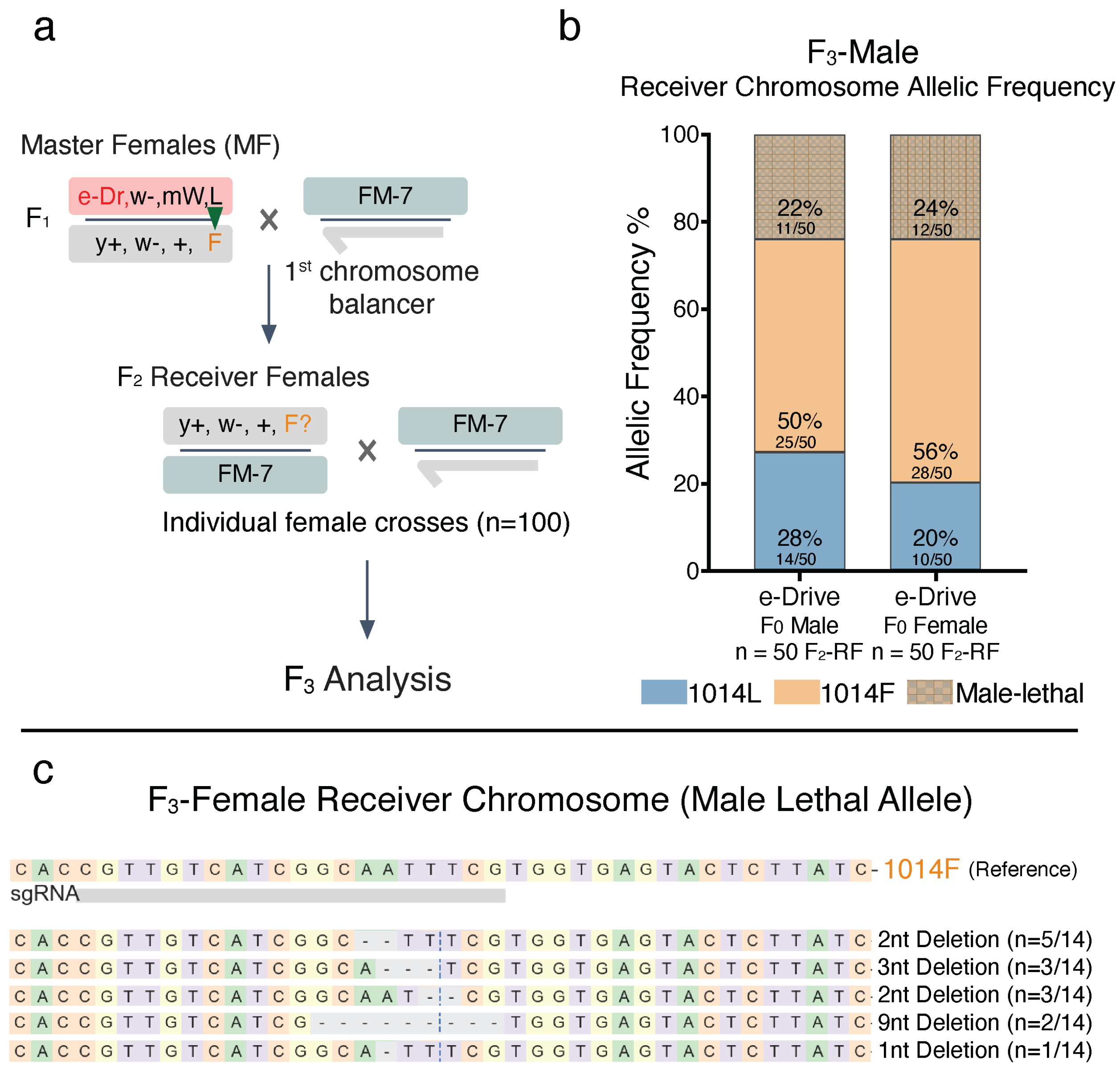

Supplementary Figure 3.

Recovery of e-Drive induced male lethal alleles from F₂ receiver females. (a) Cross scheme for recovering male lethal *para* alleles for sequence analysis (from the cross shown in Figure 2c-d). Heterozygous *e-Drive, w^-, mW, $para^{1014L}$* (donor chromosome)/*y^+, w^-, $para^{1014F}$* (receiver chromosome) F_1 females were collected and crossed to FM7 males. Individual receiver females lacking fluorescent marker and red eyes were recovered in the F_2 generation and crossed back to FM7 males to assess for the viability of the non-FM7 chromosome males. **(b)** Out of 100 individual F_2 receiver female (F_2-RF) crosses derived from either e-Drive carried by F_0 males (50 single fly crosses, left bar) or females (50 single fly crosses, right bar), total 23 lines did not yield viable

male progeny with receiver chromosome. The rest of crosses with viable receiver chromosome males carried either 1014F (49-56%) or 1014L (20-27%) alleles. **(c)** Representative data from amplicon sequencing of F_3 females carrying both the WT FM7 balancer allele and the receiver chromosome allele that is lethal in males (and homozygous lethal in females). The known WT sequence was subtracted from the combined sequence traces to yield the NHEJ sequences indicative of the specific lesions carried by each balanced female.

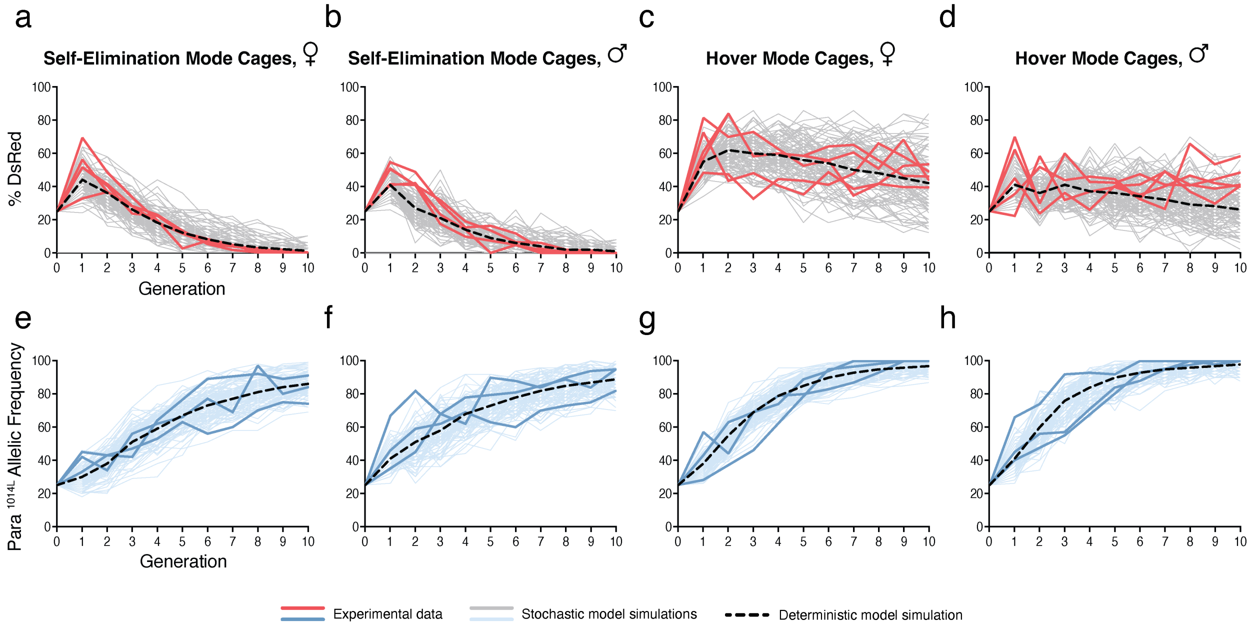

Supplementary Figure 4.

Multi-generational 1:3 ratio cage experiment and model simulation. Cages were seeded with *e-Dr,para*^{1014L} at 25% (1:3) (equal numbers of males and females), with the remaining 75% of flies carrying the insecticide resistance *para*^{1014F} allele in a *y*$^{+}$ (Self-elimination mode, Panels **a**, **b**, **e** and **f**) or *y*$^{-}$ (Hover mode, Panels **c**, **d**, **g** and **h**) background; half of progeny were used to seed the next generation. For each generation, half of the progeny were scored for prevalence of the e-Drive element (based on its DsRed^{+} marker, Panels **a-d**), and 25 randomly selected flies were sampled for DNA extraction and deep sequencing at the *para*^{1014L} locus (Panels **e-h**). Thick red and dark blue curves represent experimental data (Panels **a-d**: percentage of DsRed individuals; Panels **e-h**: percentage of WT *para*^{1014L} allele). Light grey and light blue curves represent model simulations (Panels **a-d**: percentage of DsRed individuals; Panels **e-h**: percentage of WT *para*^{1014L} allele). Black dashed curves represent deterministic model simulations (with parameters as presented in Supplementary Table 3). Grey and light blue curves represent 100 stochastic model simulations.

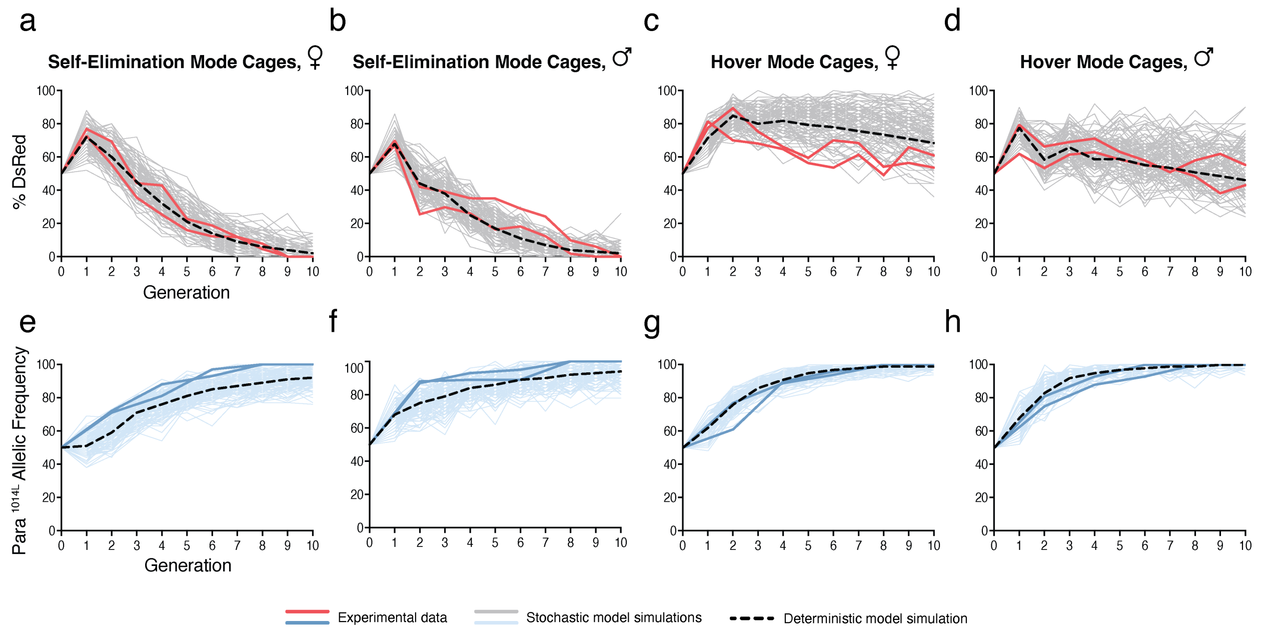

Supplementary Figure 5.

Multi-generational 1:1 ratio cage experiment and model simulation. Cages were seeded with *e-Dr,para*^{1014L} at 50% (1:1) (equal numbers of males and females), with the remaining 50% of flies carrying the insecticide resistance *para*^{1014F} allele in a y^{+} (Self-elimination mode, Panels **a**, **b**, **e** and **f**) or y^{-} (Hover mode, Panels **c**, **d**, **g** and **h**) background; half of progeny were used to seed the next generation. For each generation, half of the progeny were scored for prevalence of the e-Drive element (based on its DsRed^{+} marker, Panels **a-d**), and 25 randomly selected flies were sampled for DNA extraction and deep sequencing at the *para*^{1014L} locus (Panels **e-h**). Thick red and dark blue curves represent experimental data (Panels **a-d**: percentage of DsRed individuals; Panels **e-h**: percentage of WT *para*^{1014L} allele). Light grey and light blue curves represent model simulations (Panels **a-d**: percentage of DsRed individuals; Panels **e-h**: percentage of WT *para*^{1014L} allele). Black dashed curves represent deterministic model simulations (with parameters as presented in Supplementary Table 3). Grey and light blue curves represent 100 stochastic model simulations.

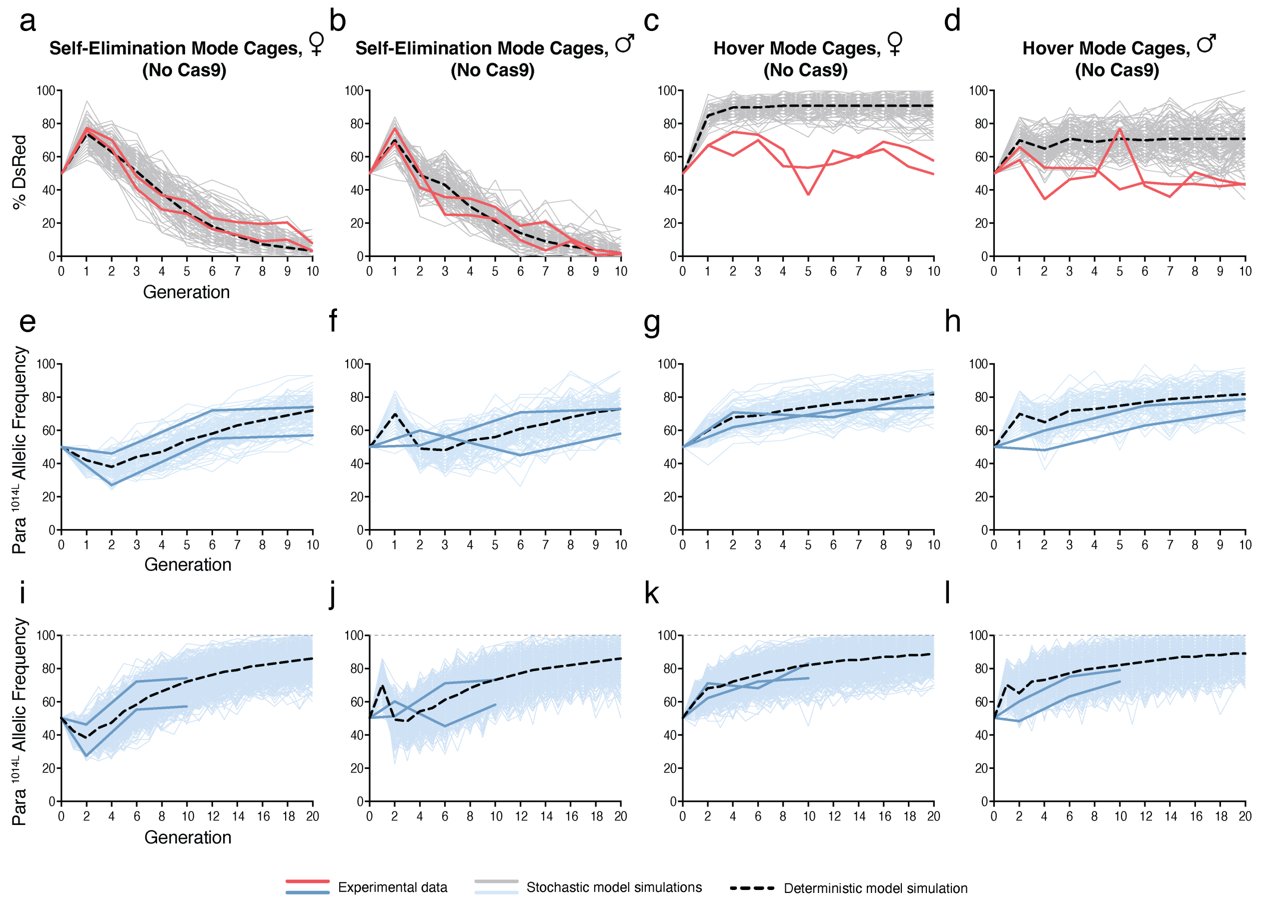

Supplementary Figure 6.

Control (no-Cas9) multi-generational 1:1 ratio cage experiment and model simulation. Control cages were seeded with *e-Dr(-Cas9),para^{1014L}* (no vasa-Cas9) at 50% (1:1) (equal numbers of males and females), with the remaining 50% of flies carrying the insecticide resistance *para^{1014F}* allele in a *y*$^{+}$ (Self-elimination mode, Panels **a**, **b**, **e**, **f**, **i** and **j**) or *y*$^{-}$ (Hover mode, Panels **c**, **d**, **g**, **h**, **k** and **l**) background; half of progeny were used to seed the next generation. For each generation, half of the progeny were scored for prevalence of the e-Drive element (-Cas9*,* based on its DsRed^{+} marker, Panels **a-d**), and 25 randomly selected flies were sampled for DNA extraction and deep sequencing at the *para^{1014F}* locus (Panels **e-h**). Thick red and dark blue curves represent experimental data (Panels **a-d**: percentage of DsRed individuals; Panels **e-l**: percentage of WT *para^{1014L}* allele up to 10 generations). Light grey and light blue curves represent model simulations (Panels **a-d**: percentage of DsRed individuals; Panels **e-h**: percentage of WT *para^{1014L}* allele up to 10 generations; Panels **i-l**: modeling the percentage of WT *para^{1014L}* allele extended through 20 generations). Black dashed curves represent deterministic model simulations (with parameters as presented in Supplementary Table 3). Grey and light blue curves represent stochastic model simulations. The *para^{1014L}* allele averaged 80-90% in both self-elimination and hover mode by generation 20. In many stochastic simulations the *para^{1014F}* allele was completely eliminated from the cages (Panels **i-l**).

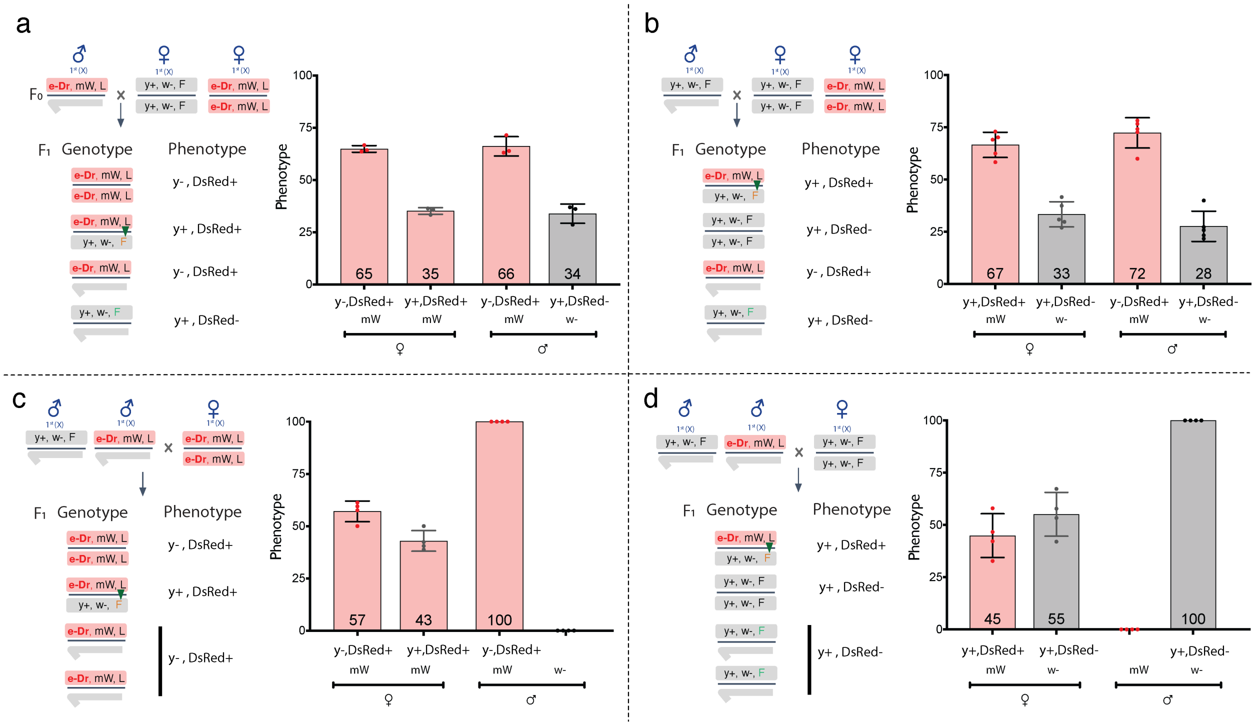

Supplementary Figure 7.

Fitness cost associated with the *para*^{1014F} allele. Competitive mating test where equal numbers of *e-Dr, para*^{1014L} and *para*^{1014F} females were crossed to either *e-Dr, para*^{1014L} (**a**, *n* = 3 independent crosses) or *para*^{1014F} males (**b**, *n* = 5 independent crosses). Reciprocal mating experiment in which equal numbers of *e-Dr, para*^{1014L} and *para*^{1014F} males were crossed to either *e-Dr, para*^{1014L} (**c**, *n* = 4 independent crosses) or *para*^{1014F} females (**d**, *n* = 4 independent crosses). Representative F$_1$-progeny phenotype data (y$^{+/–}$, DsRed $^{+/–}$) are plotted on the right. Error bars indicate mean ± standard deviation, with the mean value indicated in the bar.

Supplementary Table 1.

Simplified model framework.

Variable	Description
Y	*y*$^{+}$
C	*e-Dr*
L	*para*^{1014L}
F	*w*$^{-}$,*para*^{1014F}
N	lethal NHEJ allele at *para*^{1014F}

Supplementary Table 2.

Phenotype of flies according to the simplified model framework.

Sex	Genotype	Phenotype		
		Body	RFP	Insecticide resistance
♀	$YYLL$	brown	no fluorescence	wild type
	$YYLF$ / $YYLN$	brown	no fluorescence	partially resistant
	$YYFF$ / $YYFN$	brown	no fluorescence	resistant
	$YCLL$	brown	fluorescence	wild type
	$YCLF$ / $YCLN$	brown	fluorescence	partially resistant
	$CCLL$	yellow	fluorescence	wild type
	$CCLF$ / $CCLN$	yellow	fluorescence	partially resistant
	$YCFF$ / $YCFN$ / $YCNN$ / $YYNN$ / $CCFF$ / $CCFN$ / $CCNN$	inviable		
♂	YL	brown	no fluorescence	wild type
	YF	brown	no fluorescence	resistant
	CL	yellow	fluorescence	wild type
	CF	inviable		
	YN/CN	inviable		

Supplementary Table 3.

Model parameters, descriptions, and estimated posteriors.

Parameter	Description	Posterior [mean (95%CrI)]
r_C	rate of e-Drive conversion $para^{1014F}$ into $para^{1014L}$	$0.27\ (0.00 - 0.81)$
r_{lethal}	rate of e-Drive conversion $para^{1014F}$ into $para^{\text{lethal}}$	$0.32\ (0.00 - 0.84)$
md	rate of maternal deposition effect	$0.47\ (0.02 - 0.93)$
f_F	factor for reduced viability of $para^{1014F}$ males	Parameter excluded*
$f_{FF,viab}$	factor for reduced viability of $para^{1014F}$ homozygous females	Parameter excluded*
$f_{FF,fert}$	factor for reduced fertility of $para^{1014F}$ homozygous females	$0.44\ (0.27 - 0.63)$
$m_{\text{e-Dr}}$	mating competition factor for males carrying e-Drive system	$0.16\ (0.06 - 0.29)$
$m_{\text{e-Dr,hover}}$	mating competition factor for males carrying e-Drive system for hover mode	$0.74\ (0.60 - 0.92)$
r_C (control)	rate of e-Drive (without vasa-Cas9) conversion $para^{1014F}$ into $para^{1014L}$ in control	0
r_{lethal} (control)	rate of e-Drive (without vasa-Cas9)conversion $para^{1014F}$ into $para^{\text{lethal}}$ in control	0
md (control)	rate of maternal deposition effect in control	0
$f_{FF,fert}$ (control)	factor for reduced fertility of $para^{1014F}$ homozygous females in control	$0.44\ (0.27 - 0.63)$
$m_{\text{e-Dr}}$ (control)	mating competition factor for males carrying e-Drive (without vasa-Cas9) system in control	$0.16\ (0.06 - 0.29)$
$m_{\text{e-Dr,hover}}$ (control)	mating competition factor for males carrying e-Drive system (without vasa-Cas9) for hover mode in control	1

*See Model selection Section for details.

Supplementary Table 4.

Model selection. Here we call "standard model" the model described in the text and defined by the parameter presented in Supplementary Table 3. The best fitting model according to the AIC value is marked in green.

Model	Loglikelihood	AIC
standard model	-261.8	537.7
standard model + $m_{\mathrm{e-Dr,hover}}$	-232.5	481.0
standard model + $m_{\mathrm{e-Dr,hover}}$ - $m_{\mathrm{e-Dr}}$	-277.3	568.6
standard model + $m_{\mathrm{e-Dr,hover}}$ - f_F	-233.0	480.1
standard model + $m_{\mathrm{e-Dr,hover}}$ - $f_{FF,viab}$	-232.5	479.0
standard model + $m_{\mathrm{e-Dr,hover}}$ - $f_{FF,fert}$	-234.0	482.1
standard model + $m_{\mathrm{e-Dr,hover}}$ - md	-233.8	481.6
standard model + $m_{\mathrm{e-Dr,hover}}$ - $f_{FF,viab}$ - $m_{\mathrm{e-Dr}}$	-276.6	565.2
standard model + $m_{\mathrm{e-Dr,hover}}$ - $f_{FF,viab}$ - f_F	-232.8	477.6
standard model + $m_{\mathrm{e-Dr,hover}}$ - $f_{FF,viab}$ - $f_{FF,fert}$	-234.0	480.0
standard model + $m_{\mathrm{e-Dr,hover}}$ - $f_{FF,viab}$ - md	-233.7	479.3
standard model + $m_{\mathrm{e-Dr,hover}}$ - $f_{FF,viab}$ - f_F - $m_{\mathrm{e-Dr}}$	-276.3	562.5
standard model + $m_{\mathrm{e-Dr,hover}}$ - $f_{FF,viab}$ - f_F - $f_{FF,fert}$	-247.5	505.1
standard model + $m_{\mathrm{e-Dr,hover}}$ - $f_{FF,viab}$ - f_F - md	-234.1	478.3

Source Data. (separate file)

Raw Data and Model Fitting Data.
