# Supplementary material for: A self-eliminating allelic-drive reverses insecticide resistance in Drosophila leaving no transgene in the population
Source: Nat Commun. 2024 Nov 17;15:9961. doi: 10.1038/s41467-024-54210-4 (PMC11570635; doi:10.1038/s41467-024-54210-4)
Supplement: Supplementary file 2 — Reporting Summary [file 41467_2024_54210_MOESM2_ESM.pdf]

nature portfolio

Corresponding author(s): Ethan Bier

Last updated by author(s): Jun 4, 2024

Reporting Summary

Nature Portfolio wishes to improve the reproducibility of the work that we publish. This form provides structure for consistency and transparency in reporting. For further information on Nature Portfolio policies, see our Editorial Policies and the Editorial Policy Checklist.

Statistics

For all statistical analyses, confirm that the following items are present in the figure legend, table legend, main text, or Methods section.

n/a	Confirmed	
☐	☒	The exact sample size (*n*) for each experimental group/condition, given as a discrete number and unit of measurement
☐	☒	A statement on whether measurements were taken from distinct samples or whether the same sample was measured repeatedly
☐	☒	The statistical test(s) used AND whether they are one- or two-sided<br>*Only common tests should be described solely by name; describe more complex techniques in the Methods section.*
☒	☐	A description of all covariates tested
☒	☐	A description of any assumptions or corrections, such as tests of normality and adjustment for multiple comparisons
☐	☒	A full description of the statistical parameters including central tendency (e.g. means) or other basic estimates (e.g. regression coefficient) AND variation (e.g. standard deviation) or associated estimates of uncertainty (e.g. confidence intervals)
☐	☒	For null hypothesis testing, the test statistic (e.g. *F*, *t*, *r*) with confidence intervals, effect sizes, degrees of freedom and *P* value noted<br>*Give P values as exact values whenever suitable.*
☐	☒	For Bayesian analysis, information on the choice of priors and Markov chain Monte Carlo settings
☒	☐	For hierarchical and complex designs, identification of the appropriate level for tests and full reporting of outcomes
☒	☐	Estimates of effect sizes (e.g. Cohen's *d*, Pearson's *r*), indicating how they were calculated

Our web collection on statistics for biologists contains articles on many of the points above.

Software and code

Policy information about availability of computer code

Data collection	CRISPResso2 pipelines was used for analysis of deep sequencing results.
Data analysis	Graphs were analyzed and generated using Prism 10. Figure were made on Adobe Illustrator (2024).

For manuscripts utilizing custom algorithms or software that are central to the research but not yet described in published literature, software must be made available to editors and reviewers. We strongly encourage code deposition in a community repository (e.g. GitHub). See the Nature Portfolio guidelines for submitting code & software for further information.

Data

Policy information about availability of data

All manuscripts must include a data availability statement. This statement should provide the following information, where applicable:

- Accession codes, unique identifiers, or web links for publicly available datasets
- A description of any restrictions on data availability
- For clinical datasets or third party data, please ensure that the statement adheres to our policy

The e-Drive plasmid sequence data has been uploaded to NCBI and is available online with the accession number PP695280. All data can be found in the main text or the supplementary materials. The source data is also provided in a separate file accompanying this paper.

Research involving human participants, their data, or biological material

Policy information about studies with human participants or human data. See also policy information about sex, gender (identity/presentation), and sexual orientation and race, ethnicity and racism.

Reporting on sex and gender	N/A
Reporting on race, ethnicity, or other socially relevant groupings	N/A
Population characteristics	N/A
Recruitment	N/A
Ethics oversight	N/A

Note that full information on the approval of the study protocol must also be provided in the manuscript.

Field-specific reporting

Please select the one below that is the best fit for your research. If you are not sure, read the appropriate sections before making your selection.

☒ Life sciences ☐ Behavioural & social sciences ☐ Ecological, evolutionary & environmental sciences

For a reference copy of the document with all sections, see nature.com/documents/nr-reporting-summary-flat.pdf

Life sciences study design

All studies must disclose on these points even when the disclosure is negative.

Sample size	This study included all offspring that were produced from the given cross and population cages. However, crosses with less than 10 offspring were not included, as the low number could have been caused by contaminated food.
Data exclusions	No data was excluded in this study
Replication	We performed multiple replicates of the crosses and cage setups, resulting in the generation of over 10 offspring per cross and over 100 offsprings per cages. The replicates yielded consistent and comparable results, indicating that the experimental conditions were reliable and reproducible.
Randomization	Progenies were randomly selected from crosses and cages. We also randomly selected flies for Sanger sequencing analysis and NGS analysis.
Blinding	All fly crosses we had selection marked like white +, yellow+, or dsRed in eyes to identify appropriate genotype of progenies. Investigators were not blinded with all these scoring genotypes.

Reporting for specific materials, systems and methods

We require information from authors about some types of materials, experimental systems and methods used in many studies. Here, indicate whether each material, system or method listed is relevant to your study. If you are not sure if a list item applies to your research, read the appropriate section before selecting a response.

Materials & experimental systems

n/a	Involved in the study	
☒	☐	Antibodies
☒	☐	Eukaryotic cell lines
☒	☐	Palaeontology and archaeology
☐	☒	Animals and other organisms
☒	☐	Clinical data
☒	☐	Dual use research of concern
☒	☐	Plants

Methods

n/a	Involved in the study	
☒	☐	ChIP-seq
☒	☐	Flow cytometry
☒	☐	MRI-based neuroimaging

Animals and other research organisms

Policy information about studies involving animals; ARRIVE guidelines recommended for reporting animal research, and Sex and Gender in Research

Laboratory animals	Drosophila melanogaster strain and age of flies are reported in this study.
Wild animals	No wild animals were used in this study.
Reporting on sex	Sex and genotype of flies are reported in this study.
Field-collected samples	No field collection was carried out.
Ethics oversight	All fly crosses were carried out in glass vials in ACL-1 facility

Note that full information on the approval of the study protocol must also be provided in the manuscript.
